# Genetic associations vary across the spectrum of fasting serum insulin: results from the European IDEFICS/I.Family children’s cohort

**DOI:** 10.1007/s00125-023-05957-w

**Published:** 2023-07-07

**Authors:** Kirsten Mehlig, Ronja Foraita, Rajini Nagrani, Marvin N. Wright, Stefaan De Henauw, Dénes Molnár, Luis A. Moreno, Paola Russo, Michael Tornaritis, Toomas Veidebaum, Lauren Lissner, Jaakko Kaprio, Iris Pigeot, on behalf of the I.Family consortium

**Affiliations:** 1 School of Public Health and Community Medicine, Institute of Medicine, University of Gothenburg, Gothenburg, Sweden; 2 Leibniz Institute for Prevention Research and Epidemiology – BIPS, Bremen, Germany; 3 Department of Mathematics and Computer Science, University of Bremen, Bremen, Germany; 4 Department of Public Health, University of Copenhagen, Copenhagen, Denmark; 5 Department of Public Health and Primary Care, Faculty of Medicine and Health Sciences, Ghent University, Ghent, Belgium; 6 Department of Paediatrics, Medical School, University of Pécs, Pécs, Hungary; 7 GENUD (Growth, Exercise, Nutrition and Development) Research Group, University of Zaragoza, Zaragoza, Spain; 8 Instituto Agroalimentario de Aragón (IA2), Zaragoza, Spain; 9 Instituto de Investigación Sanitaria de Aragón (IIS Aragón), Zaragoza, Spain; 10 Centro de Investigación Biomédica en Red de Fisiopatología de la Obesidad y Nutrición (CIBERObn), Instituto de Salud Carlos III, Madrid, Spain; 11 Institute of Food Sciences, National Research Council, Avellino, Italy; 12 Research and Education Institute of Child Health, Strovolos, Cyprus; 13 National Institute for Health Development, Tallinn, Estonia; 14 Institute for Molecular Medicine Finland, University of Helsinki, Helsinki, Finland

**Keywords:** Biomarkers, BMI, Dementia, Genetics, Genome-wide association analysis, Insulin, Metabolic traits, Obesity, Quantile regression, SNP, Type 2 diabetes

## Abstract

**Aims/hypothesis:**

There is increasing evidence for the existence of shared genetic predictors of metabolic traits and neurodegenerative disease. We previously observed a U-shaped association between fasting insulin in middle-aged women and dementia up to 34 years later. In the present study, we performed genome-wide association (GWA) analyses for fasting serum insulin in European children with a focus on variants associated with the tails of the insulin distribution.

**Methods:**

Genotyping was successful in 2825 children aged 2–14 years at the time of insulin measurement. Because insulin levels vary during childhood, GWA analyses were based on age- and sex-specific *z* scores. Five percentile ranks of *z*-insulin were selected and modelled using logistic regression, i.e. the 15th, 25th, 50th, 75th and 85th percentile ranks (P15–P85). Additive genetic models were adjusted for age, sex, BMI, survey year, survey country and principal components derived from genetic data to account for ethnic heterogeneity. Quantile regression was used to determine whether associations with variants identified by GWA analyses differed across quantiles of log-insulin.

**Results:**

A variant in the *SLC28A1* gene (rs2122859) was associated with the 85th percentile rank of the insulin *z* score (P85, *p* value=3×10^−8^). Two variants associated with low *z*-insulin (P15, *p* value <5×10^−6^) were located on the *RBFOX1* and *SH3RF3* genes. These genes have previously been associated with both metabolic traits and dementia phenotypes. While variants associated with P50 showed stable associations across the insulin spectrum, we found that associations with variants identified through GWA analyses of P15 and P85 varied across quantiles of log-insulin.

**Conclusions/interpretation:**

The above results support the notion of a shared genetic architecture for dementia and metabolic traits. Our approach identified genetic variants that were associated with the tails of the insulin spectrum only. Because traditional heritability estimates assume that genetic effects are constant throughout the phenotype distribution, the new findings may have implications for understanding the discrepancy in heritability estimates from GWA and family studies and for the study of U-shaped biomarker–disease associations.

**Graphical Abstract:**

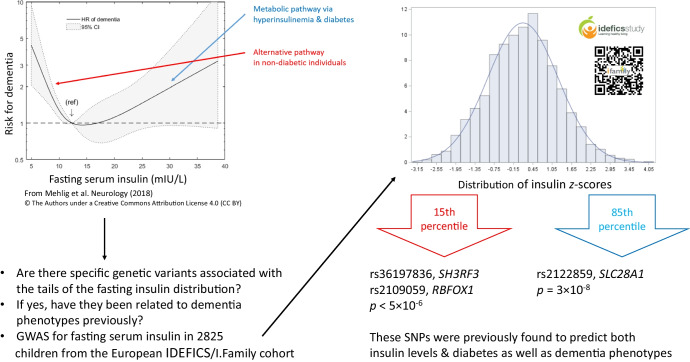

**Supplementary Information:**

The online version contains peer-reviewed but unedited supplementary material available at 10.1007/s00125-023-05957-w.


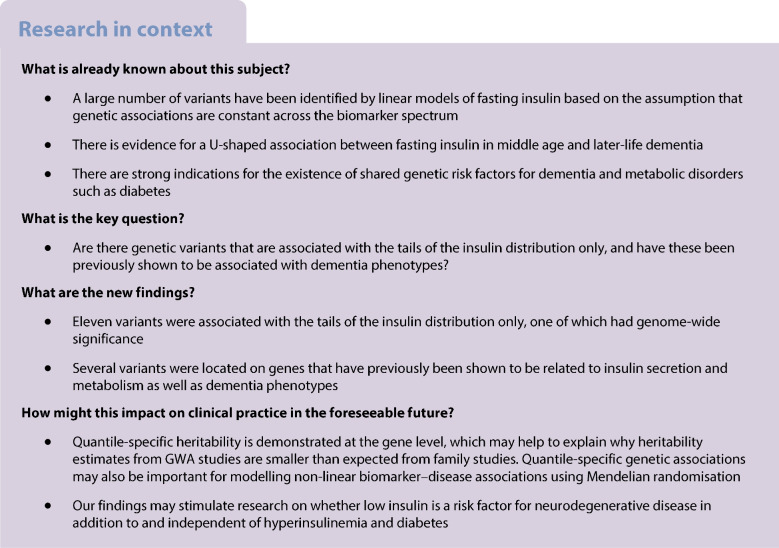


## Introduction

Fasting serum insulin is an important marker for metabolic disorders, including obesity, type 2 diabetes and the metabolic syndrome. Because these conditions run in families [1], genome-wide association (GWA) studies have been performed to assess fasting insulin, hyperinsulinaemia or diabetes [2–8]. These analyses are usually performed in adults, even though type 2 diabetes may be observed in childhood [9]. Gene/biomarker data for children are particularly valuable because it may be assumed that children’s biomarker values are less influenced by acquired lifestyle than those in adults. While GWA studies on children are still rare, this project aimed to identify genetic determinants of fasting insulin in a population-based sample of children in Europe below the age of 15 years. Specifically, we performed GWA analyses for selected percentiles of the insulin distribution to test whether the association pattern differed towards the low and high ends of the insulin spectrum. This was motivated by observations of a U-shaped relationship between fasting insulin in middle age and dementia up to 34 years later [10]. Women in the lowest tertile of fasting insulin showed a higher prevalence of the *APOE-4* allele compared to women with higher insulin, which is remarkable as the *APOE-4* allele is the strongest genetic risk factor for dementia, particularly Alzheimer’s disease [11]. Other variants are expected to be associated with hyperinsulinaemia, many of which have already been identified because of their association with type 2 diabetes. Type 2 diabetes per se has often been found to be associated with dementia, in particular among non-carriers of the *APOE-4* allele [10, 12, 13]. Taken together, these findings support the idea that genetic variants differentially associated with the two extremes of the insulin spectrum imply the existence of different disease mechanisms for cognitive impairment and dementia. The demonstration that genetic associations vary across the insulin spectrum may have implications for the analysis of U-shaped biomarker–disease associations using Mendelian randomisation in general.

## Methods

### Study participants

The European IDEFICS (Identification and prevention of Dietary- and lifestyle-induced health EFfects In Children and infantS)/I.Family cohort is a multi-centre population-based children’s study that aimed to identify risk factors for diet- and lifestyle-related diseases, with a focus on childhood overweight and metabolic disorders [14, 15]. Children were recruited through kindergarten or school settings in Belgium, Cyprus, Estonia, Germany, Hungary, Italy, Spain and Sweden. In each country, two or more communities with similar sociodemographic profile and infrastructure were selected, which were typical for their region but not for the survey country as a whole. In 2007/2008, 16,229 children aged between 2 and 9.9 years participated in the baseline survey. Follow-up surveys were conducted after 2 years (*n*=11,043, plus 2543 newcomers [recruited from different families within the same community]) and 6 years (*n*=7117, plus 2512 newly recruited siblings). Children were asked to provide fasting venous samples, morning urine samples and saliva samples. If consent for venous blood withdrawal was not given, capillary blood was taken with the consent of the children and parents. The study was conducted in agreement with the Declaration of Helsinki; all procedures were approved by the local ethics committees, and written and oral informed consents were obtained. The IDEFICS/I.Family cohort study is registered in the ISRCTN clinical trial registry (https://doi.org/10.1186/ISRCTN62310987).

### Genotyping and biomarker assessment

Children were selected for a whole-genome scan based on their participation in the individual study modules (children who had participated in more examinations within the study were prioritised) [16]. Children from Cyprus were not included in the initial genotyping. DNA was extracted from saliva or blood samples of 3515 children. Genotyping was performed on the UK Biobank Axiom 196-Array (Affymetrix, USA), which, after quality control and imputation, resulted in 3,424,677 variants for 3099 children [16]. Association results are given for the minor allele at each locus (effect allele). Haplotypes of apolipoprotein E (*APOE*) were determined by direct genotyping of rs429358 and rs7412, and the number of *APOE-4* alleles was calculated. Genetically determined sex was used as a confounder in regression analyses. Fasting serum insulin was measured using an electrochemiluminescence immunoassay (Roche, Germany). Further details on laboratory and genetic analyses have been published previously [14–18]. Insulin levels vary considerably during childhood and adolescence, with a pronounced rise before and during puberty [19]. Using data from the IDEFICS cohort, this rise was confirmed by Peplies et al, who computed age- and sex-specific percentiles and *z* scores for children up to 11 years old [17]. Insulin percentiles and *z* scores for children up to the age of 14 years were added later using data from the second follow-up survey [20]. To account for developmental differences in insulin values, we selected five values for age- and sex-specific insulin percentile ranks as outcome variables for our GWA analyses. Specifically, we chose the 15th and 85th percentile ranks to describe ‘low and high for age and sex’ insulin values, respectively. These were the most extreme percentile ranks that allowed for well-defined logistic models given the number of cases in relation to the total number of predictors. For comparison, we also performed GWA analyses for the quartiles of *z*-insulin, i.e. the 25th, 50th (median) and 75th percentile ranks. Measured weight and height were used to calculate BMI, as well as age- and sex-specific *z* scores derived from the subsample of children with normal weight status according to international references [21].

### Definition of the analytical sample

Among the 3099 children for whom genetic data was available, 2825 had at least one value for fasting serum insulin at baseline, first or second follow-up (5416 observations). Restriction to the earliest measurement resulted in 2825 unique observations from the same number of children aged 2.2–14.8 years (50% female). All children had been fasting for at least 8 h before blood withdrawal, and none had a diagnosis of diabetes or took glucose-lowering medication [17].

### Statistical methods

Values for basic characteristics of the analytical sample were calculated as means (SD) for continuous variables and frequencies (%) for discrete variables. The distribution for fasting insulin was positively skewed, and the logarithm of insulin was calculated to achieve an approximately normal distribution. Five age- and sex-specific percentile ranks (P15, P25, P50, P75, P85) were selected as outcomes for logistic GWA analyses. For P15 and P25, we compared cases with *z*-insulin values at the respective percentile rank or below with all observations of higher values of *z*-insulin. For the median percentile rank (P50), as well as P75 and P85, cases with *z*-insulin values higher than or equal to the respective percentile rank were compared to all observations with lower values of *z*-insulin. We used additive genetic models adjusting for age, age^2^, sex, survey, country and 32 principal components, using the latter to account for population stratification in this heterogeneous sample of children from seven European countries. We further adjusted for BMI as insulin levels are strongly influenced by weight status. BMI was used instead of the BMI *z* score because the respective regression models showed higher values for the coefficient of determination (*R*^2^).

**Fig. 1:**
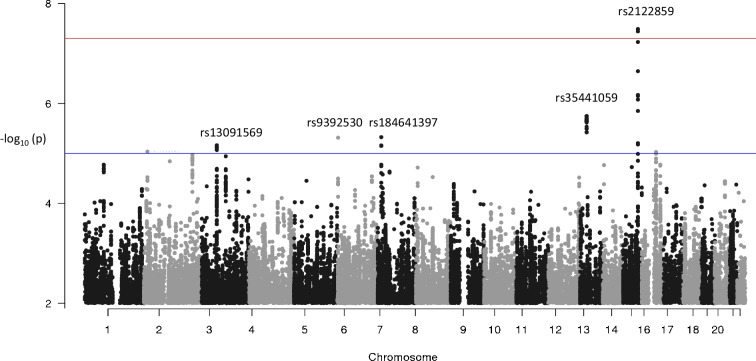
Manhattan plot illustrating GWA analysis results for the 85th percentile rank of the age- and sex-specific insulin distribution, indicating rs-numbers for SNPs with associations with *p*<10^−5^ (blue line) or *p*<5×10^−8^ (red line)

To examine how associations with individual SNPs identified through GWA analyses differed across the spectrum of log-insulin, we performed quantile regression for quantile levels between 0.05 and 0.95, with a step size of 0.05, and tested whether effect estimates for individual variants differed across quantile levels (Wald test for heteroscedasticity). Regression parameters were estimated using the simplex algorithm, and the sparsity method was used to calculate confidence intervals. Quantile process plots illustrated the variation of regression parameters across the spectrum of log-insulin. For each quantile level, the adjusted coefficient of determination (*R*^2^_adj_) was calculated using the SAS macro quant_gof [22]. We also present effect estimates for variants identified in the logistic GWA analyses using linear regression for log-insulin. Sensitivity analyses addressed the influence of between-sibling correlations in linear mixed models including family as random effect. Although GWA analyses identified SNPs associated with age- and sex-specific percentile ranks, further evaluation was based on log-insulin to facilitate comparison with effect size measures reported in previous publications. The sequence of analyses is illustrated in electronic supplementary material [ESM] Fig. 1.

For GWA analyses, we used a genome-wide statistical significance level of 5×10^−8^, and 10^−5^ for suggestive significant associations [23]. Because the quantile and linear regression analyses of log-insulin and individual SNPs consisted of 25 independent tests, we adopted a Bonferroni-corrected significance level of 0.05/25=0.002 when reporting these results. GWA analyses were performed using PLINK version 1.90b3.42 [24] (https://www.cog-genomics.org/plink2) and R software version 3.4.3 (https://cran.r-project.org/bin/windows/base/old/). All other analyses were performed using SAS version 9.4 (https://support.sas.com/software/94/).

## Results

Table 1 provides the basic characteristics of the analytical sample. The mean age of the children was 7 years, with an age range from 2.2 to 14.8 years, and 50% were girls. The proportions of girls did not vary across the age range. A mean BMI *z* score larger than zero shows that the genetic sample included children with overweight and obesity, who had not been part of the reference population of normal weight children [25]. This is also the reason why the mean values for percentile rank and *z*-insulin exceed 50% and 0.0, respectively. The distribution of fasting insulin was skewed to the right, with skewness = 4.3 and a maximum insulin value of 451 pmol/l (65 mIU/l). The 33 children with fasting insulin >138 pmol/l (20 mIU/l) had higher mean values for age, plasma glucose and BMI than those with lower insulin values, and 26 of them had extreme obesity (BMI *z* score=5), but there were no differences by sex. Because none of these children were diagnosed with diabetes at the time of blood sampling, and their insulin values were not outliers on logarithmic scale, their observations were included in the analytical sample. Fasting serum insulin was positively correlated with age and BMI (*r* values=0.38 and 0.54, respectively). For children participating in more than one examination, the first valid insulin measurement was selected, i.e. at the youngest age. Overall, most of the measurements included in this study were taken at baseline, and 31% were obtained at follow-up examinations.

**Table 1:** Basic characteristics of the insulin sample used for GWA analyses (*n*=2825)

Variable	Value	Range
Age (years)	7.2 (2.3)	2.2, 14.8
BMI (kg/m^2^)	16.9 (3.1)	10.2, 35.8
BMI *z* score	0.6 (2.9)	−5.0, 5.0
Insulin (pmol/l)	35.0 (29.7)	0.2, 451.5
Insulin *z* score [17]	0.14 (1.09)	−3.1, 4.1
Percentile rank (%)	53.7 (30.2)	0.11, 99.9
Female, *n* (%)	1411 (50)	
Survey country, *n* (%)		
Belgium	194 (7)	
Estonia	277 (10)	
Germany	573 (20)	
Hungary	426 (15)	
Italy	598 (21)	
Spain	359 (13)	
Sweden	398 (14)	
Survey, *n* (%)		
Baseline examination (2007/2008)	1958 (69)	
1st follow-up (2009/2010)	611 (22)	
2nd follow-up (2013/2014)	256 (9)	

Values for continuous variables are presented as means (SD) as well as range (min, max); values for categorical variables are presented as *n* (%)

### GWA analysis results for selected percentiles of the insulin distribution

Table 2 shows the results for SNPs identified in logistic GWA analyses of the selected insulin percentile ranks (*p*<10^−5^). Genetic associations were determined with all five percentile ranks, with a comparable effect size but higher statistical significance at the high end of the insulin spectrum. For instance, the association between a variant on the *SLC28A1* gene and the 85^th^ percentile rank reached genome-wide significance. A variant on the *RAPGEF4* gene was associated with the 75th percentile rank at *p*=5.1×10^−8^. The majority of SNPs were located on genes with known function in insulin secretion, metabolism or clearance. The effect allele frequencies agreed well with frequencies reported for participants of European descent (using dbSNP, www.ncbi.nlm.nih.gov/snp/). Manhattan plots illustrate GWA analysis results for the 85th percentile rank (Fig. 1) and the 15th–75th percentile ranks (ESM Fig. 2). A posteriori logistic regression models showed that the associations between specific SNPs and percentiles of the insulin distribution shown in Table 2 did not differ by sex (data not shown).

**Table 2:** GWA analysis results for selected age- and sex-specific percentiles of fasting insulin (*n*=2825)^a^

Percentile rank^b^	SNP	Chromosome	Position (GRCh37)	Reference/EA^c^	EAF	OR	SE (log OR)	*p*	Nearest gene	*p*_hs_^d^	Linear effect size (SE)^e^
P15 (*n*=402)	rs36197836	2	110252614	G/A	0.228	0.61	0.11	4.7 ×10^−6^	*SH3RF3* [28]	0.003	0.051 (0.019)
	rs4855039	3	181470951	T/A	0.413	0.68	0.08	5.2 ×10^−6^		<0.0001	0.066 (0.016)^*^
	rs34988275	5	3549238	C/T	0.060	2.03	0.15	1.7 ×10^−6^		0.08	−0.091 (0.034)
	rs7896849	10	3798495	C/A	0.348	1.46	0.08	5.7 ×10^−6^	*LOC105376361*	0.06	−0.063 (0.016)^*^
	rs61842989	10	28443235	A/G	0.067	1.91	0.14	7.4 ×10^−6^	*MPP7* [41]	0.01	−0.073 (0.032)
	rs2109059	16	7709185	G/A	0.234	1.57	0.09	9.4 ×10^−7^	*RBFOX1* [27]	0.0003	−0.040 (0.019)
P25 (*n*=644)	rs841528	6	65899528	G/A	0.313	0.70	0.08	2.1 ×10^−6^	*EYS*	0.0004	0.050 (0.017)
	rs8110900^f^	19	53788609	G/C	0.072	1.80	0.13	4.6 ×10^−6^		0.009	−0.085 (0.031)
	rs926276	6	96868735	T/C	0.207	1.45	0.08	8.3 ×10^−6^	*UFL1-AS1*	0.004	−0.052 (0.020)
	rs11243014	6	5511614	G/A	0.115	0.59	0.12	9.6 ×10^−6^	*FARS2* [42]	0.06	0.053 (0.025)
P50 (*n*=1570)	rs518352 ^g^	1	89740719	G/A	0.273	1.36	0.07	6.2 ×10^−6^	*GBP5*	0.01	0.056 (0.018)
	rs1708231	3	129675157	C/T	0.177	0.68	0.08	1.0 ×10^−6^		0.2	−0.088 (0.021)^*^
	rs112477351^h^	5	138553304	T/C	0.063	1.78	0.12	3.0 ×10^−6^	*SIL1* [43]	0.01	0.087 (0.032)
	rs75530245	6	87693976	T/C	0.077	1.74	0.12	4.8 ×10^−6^	*HTR1E*	0.03	0.121 (0.030)^*^
	rs12339899	9	127677918	C/T	0.125	0.67	0.09	9.7 ×10^−6^	*GOLGA1*	0.07	−0.088 (0.024)^*^
P75 (*n*=877)	rs2305462	2	173895285	G/A	0.153	1.61	0.09	5.1 ×10^−8^	*RAPGEF4* [44, 45]	0.5	0.102 (0.022)^*^
	rs76532059	3	25475980	G/C	0.069	1.74	0.12	5.8 ×10^−6^	*RARB* [46, 47]	0.2	0.092 (0.031)
	rs184641397	7	14358575	T/C	0.093	1.71	0.11	4.7 ×10^−7^	*DGKB* [48]	0.15	0.083 (0.027)
	rs12909687	15	51372071	A/G	0.227	1.42	0.08	5.7 ×10^−6^	*TNFAIP8L3* [49], *MIR4713HG*	0.15	0.071 (0.019)^*^
	rs413285	21	16611303	A/G	0.490	0.71	0.07	2.2 ×10^−7^		0.3	−0.039 (0.016)
P85 (*n*=573)	rs13091569	3	63630271	A/C	0.139	1.59	0.10	6.8 ×10^−6^		0.02	0.048 (0.023)
	rs9392530	6	3842866	C/T	0.481	1.42	0.08	4.8 ×10^−6^	*FAM50B* [50]	0.05	0.042 (0.016)
	rs184641397	7	14358575	T/C	0.093	1.74	0.12	4.7 ×10^−6^	*DGKB* [48]	0.15	0.083 (0.027)
	rs35441059	13	47095150	C/T	0.155	1.61	0.10	1.8 ×10^−6^		0.003	0.036 (0.022)
	rs2122859	15	85470292	G/T	0.185	1.68	0.09	3.3 ×10^−8^	*SLC28A1* [26]	0.01	0.078 (0.020)^*^

^a^Genome-wide logistic regression adjusted for age, sex, BMI, country and principal components

^b^Numbers represent the numbers of observations below P15 and P25, and the numbers of observations above P50, P75 and P85

^c^Reference/effect allele (EA)

^d^*p* value for heteroscedasticity across quantiles; after correction for multiple testing, results with *p* values <0.002 were considered significant

^e^Beta coefficient from linear regression of log-insulin on genotype adjusted for age, age^2^, sex, BMI, country and principal components; after correction for multiple testing, results with *p* values <0.002 were considered significant (as indicated by asterisks)

^f^Linkage disequilibrium with rs7412 (*APOE-4*): *r*^2^=0.058, *D*ʹ=0.999

^g^The closely linked variant rs547544 (OR=1.36, *p*=6.4×10^−6^) is located on the *GBP5* gene

^h^The closely linked variant rs113278654 (OR=1.79, *p*=5.5×10^−6^) is located on the *SIL1* gene

EAF, effect allele frequency

### Quantile regression to test the variability of associations with selected SNPs across the insulin spectrum

**Fig. 2:**
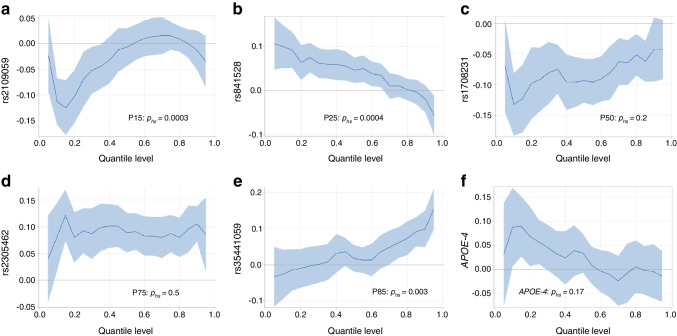
Quantile regression plots for selected SNPs identified by GWA analyses of insulin percentile ranks P15 (**a**), P25 (**b**), P50 (**c**), P75 (**d**) and P85 (**e**) (Table 2) as well as for the *APOE-4* genotype (**f**), including a test for heteroscedasticity across quantile levels (*p*_hs_ value). Quantile regression of log-insulin was performed for selected SNPs and adjusted for age, age^2^, sex, BMI, survey, country and principal components (regression parameters with 95% confidence bands). Regression models for *APOE-4* were not further adjusted for BMI

Table 2 also shows the *p* value for tests of heteroscedasticity, which indicates to what extent the association with a certain variant varies across quantiles of log-insulin. After adjustment for multiple testing, only SNPs associated with low percentile ranks (P15 and P25) showed significant variation across quantiles. The variation of allele-specific associations across the insulin spectrum is further illustrated by quantile process plots, which are shown in Fig. 2 for selected SNPs associated with the five percentile ranks of *z*-insulin. Quantile process plots for all SNPs are given in the ESM Fig. 3. Variants associated with P15 and P25 showed non-zero effect sizes below the median quantile, and reduced or zero effect sizes at the high end of the spectrum, while those associated with the 85th percentile showed larger absolute effect sizes for higher quantile levels. In contrast, variants associated with P50 and P75 showed stable associations across insulin quantiles (i.e. these were not significant at the Bonferroni-adjusted significance level of 0.002). The furthest right column of Table 2 shows that several variants identified in GWA analyses for insulin percentiles were also significantly associated with log-insulin in linear regression analyses.

**Fig. 3:**
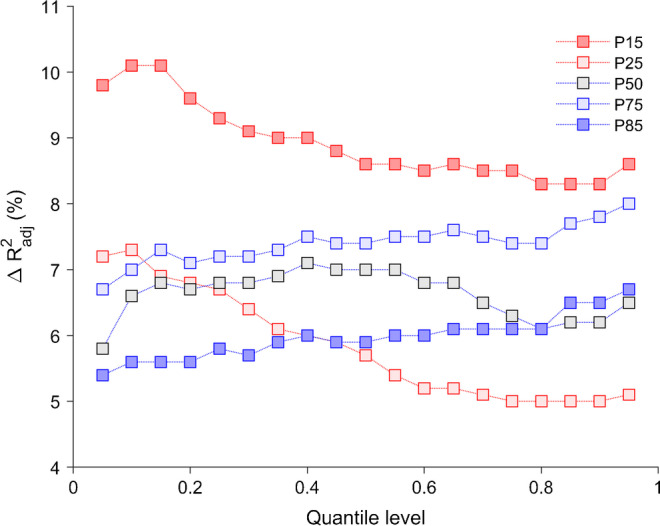
Improvement in coefficient of determination for quantile models of log-insulin adjusted for SNPs identified in GWA analyses for insulin percentiles P15, P25, P50, P75 and P85 relative to a model without genetic predictors. Quantile regression of log-insulin was performed for groups of SNPs and adjusted for age, sex, BMI, survey, country and principal components. Adjusted *R*^2^ (*R*^2^_adj_) calculated as described by Koenker and Machado [22]

To compare the prediction of log-insulin by the five sets of genetic variants, we computed the adjusted coefficient of determination (*R*^2^) generalised to quantile regression [22]. Figure 3 shows the model improvement compared to regression models without genetic predictors (Δ*R*^2^) for quantile levels between 0.05 and 0.95. The largest improvement was obtained by adding variants that predicted the lowest percentile rank of fasting serum insulin (P15). Those variants explained up to 10% of the variation at the 0.05 quantile level of log-insulin and 8.5% at the 0.95 quantile level. Δ*R*^2^ increased with higher quantile levels when variants associated with P75 and P85 were included in the model, while Δ*R*^2^ was approximately constant for variants associated with P50. The approximately U-shaped variation of *R*^2^ across quintiles of log-insulin was also displayed by regression models without genetic predictors, and the improvement in *R*^2^ by adding all genetic predictors decreased from 28.8% at quantile 0.05 to 27.3% at quantile 0.95 (ESM Table 1).

Furthermore, quantile regression of *APOE-4* showed a positive per-allele effect on log-insulin in quantile models not adjusted for BMI (Fig. 2), but this association was no longer observed upon adjustment for BMI (ESM Fig. 3). We also attempted to replicate previously published genetic associations with log-insulin (ESM Table 2). Five variants showed linear associations with log-insulin, but hardly any variation across quantile levels (ESM Fig. 4). Only 9% of the children in this study were siblings. Linear mixed models including family as a random effect did not reduce the associations reported in Table 2 (data not shown).

## Discussion

We performed GWA analyses for fasting serum insulin in children from seven European countries, with a special focus on the identification of variants associated with the tails of the fasting serum insulin distribution. Separate regression models for insulin values that were low or high for age and sex (insulin *z* scores) yielded different sets of SNPs that showed characteristic variation of effect size across quantile levels of log-insulin. Variants associated with the highest values of age- and sex-specific insulin showed associations with above-median values of log-insulin but not with values below the median, and were often located in genes that had been found to be related to type 2 diabetes in previous studies. For instance, a variant associated with the 85th percentile of *z*-insulin was located on the *SLC28A1* (or *CNT1*) gene that has been linked to type 2 diabetes [26]. Two variants associated with the 15th percentile of *z*-insulin were located on genes previously linked with insulin secretion and beta cell function (*RBFOX1*) [27] and measures of insulin resistance (*SH3RF3*) [28]. While most associations with individual variants were quantile-specific, we found that the total genetically explained variance of fasting insulin varied between 28.8% at the 0.05 quantile and 27.3% at the 0.95 quantile level of log-insulin.

The use of a population-based sample of children up to the age of 14 years is a strength of the study that allows investigation of genetic associations with insulin at a time when lifestyle factors such as smoking do not yet play a major role. The limited sample size is the main limitation of this study. While the aim of this work was mainly as proof of concept, it may stimulate replication studies in larger cohorts, not least genotyping of the entire IDEFICS/I.Family cohort comprising stored blood samples for up to 20,000 children. The large ethnic variety of participants may be both a strength and a limitation. On the one hand, it offers a wide range of potential risk variants; on the other hand, the heterogeneity reduces the statistical power. In view of the pulsative nature of insulin secretion, the lack of a second insulin measurement is also a major limitation. A second measurement would also have been preferable for children with high insulin values, although hyperinsulinaemia is common among children with obesity [29, 30]. Furthermore, childhood and adolescence are characterised by large hormonal changes, including rising insulin values and insulin resistance during puberty. Due to incomplete information, it was not possible to adjust for pubertal status; however, research showing that age is a better predictor of juvenile insulin resistance than direct measures of pubertal status [19] suggests that the age-adjustment used here may have been sufficient. The inclusion of insulin measurements from various survey examinations may cause bias due to methodological differences that are not accounted for by adjustment for year of examination. However, methods were carefully harmonised across survey examinations and countries, and no effect modification by survey was observed (data not shown). Finally, it is known that physical activity reduces insulin levels and risk of insulin resistance independently of weight status. Due to the lack of consistent measures of physical activity across surveys, we did not adjust for physical activity other than indirectly via BMI.

In 2012, Williams coined the term ‘quantile-specific expressivity’ to describe the dependence of genetic effects on the level of a phenotype, and proposed that this may be due to genes affecting concentration-dependent enzymatic reactions [31]. He used family data to show that heritability varied across quantiles for a number of biomarkers, including metabolic traits and insulin [32]. To our knowledge, this is the first study assessing quantile-specific gene–biomarker associations based on genetic variants themselves. However, there is an important difference in results between the two approaches, as Williams reports an increasing heritability across the spectrum of fasting insulin, which is not replicated in this study. One explanation may be that we focused on single variants associated with selected percentiles of the insulin distribution, while the family-based heritability estimates include the entire set of genetic determinants. Second, we observed that variants associated with the 15th percentile rank explained a larger proportion of variability of log-insulin than those associated with higher percentiles, but the latter showed higher statistical significance and less variation across the spectrum, suggesting that their combined effect on insulin may be larger. It is also possible that environmental factors such as cigarette smoking and the low body weight associated with it confound the heritability results at low insulin levels obtained in the Framingham Heart study [32], which includes individuals of age 16 or older, but the question requires further investigation. A family-based study of Finnish twins showed that additive genetic effects on BMI decreased across trait values [33]. These results give some support to the results presented here, given the positive correlation between BMI and fasting serum insulin.

Our study is one of the first and largest GWA studies of fasting insulin in children, with the youngest participants. A previous study on 679 Chilean adolescents aged 16–17 years [34] identified a novel variant in the *CSMD1* gene (rs77465890, chromosome 8) with genome-wide significance, and several suggestive associations. The variant rs77465890 was not available in the IDEFICS/I.Family cohort; however, it has been shown to be in linkage disequilibrium with a variant associated with the median percentile (P50, rs62511932;, *r*^2^=0.07, *D*ʹ=0.999, using dbSNP). More than 70 loci have been identified by previous GWA studies of fasting insulin in adults [2–5]. The associations for five loci were reproduced in the present children’s study. These SNPs showed hardly any variation across quantiles, as expected for variants identified in GWA studies using linear regression.

The main motivation for this study came from the observation of a U-shaped risk curve for fasting serum insulin and incident dementia in adults, which suggested that different genetic variants may be associated with the tails of the insulin spectrum and the various phenotypes of dementia [10]. Consistent findings of an association between diabetes and dementia prompted researchers to investigate the shared genetic architecture between metabolic traits and Alzheimer’s disease [35] and other neuropsychiatric disorders [36]. The present study showed that a variant associated with the high end of the *z*-insulin spectrum (rs2122859) was located on the *SLC28A1* gene, which has been shown to be associated with both diabetes [26] and late-onset Alzheimer’s disease [37]. Interestingly, two variants associated with low *z*-insulin in the present study were located on *RBFOX1* and *SH3RF3*, genes that have previously been shown to be related to brain amyloidosis in preclinical Alzheimer’s disease [38] and to late-onset Alzheimer’s disease [39], respectively. The lack of sex-specific genetic effects is of interest in this connection. Because most of the children in this sample were pre-pubertal, it may be suggested that the sex differences in risk for dementia in adults are strongly related to female sex hormones, particularly oestrogen. Regarding the previous observation of higher *APOE-4* allele prevalence in non-diabetic women with low insulin levels compared to those with medium or high insulin levels [10], we confirmed that the e4 allele was exclusively associated with the low end of the insulin spectrum in the children’s study; however, the effect size was small and was further reduced upon adjustment for BMI. Future studies may wish to test associations between dementia and genetic variants associated with the low end of the insulin spectrum to establish whether the extremes of the insulin spectrum are indeed related to different pathways and phenotypes of dementia as suggested by the U-shaped risk curve reported previously [10].

In summary, this study presents evidence for the notion of quantile-specific heritability based on individual genetic variants. The use of children’s data reduces the impact of environmental factors that may suggest an alternative explanation on the basis of gene–environment interaction. Our findings may help to explain the fact that heritability estimates from GWA studies are smaller than expected from family studies. Quantile-specific genetic associations may also prove important for Mendelian randomisation studies that aim to model non-linear phenotype-disease associations [40]. Inclusion of variants with quantile-specific associations may allow modelling of different parts of the biomarker spectrum independently, something that is harder to achieve using variants with a constant association across the biomarker spectrum in question.

## Supplementary Information

Below is the link to the electronic supplementary material.

Supplementary file1 (PDF 1161 KB)

## Data Availability

Due to the fact that the dataset comprises highly sensitive data collected in children, ethical restrictions prohibit the authors from making the dataset publicly available. However, data are available from the authors upon reasonable request and with permission of the steering committee on a case-by-case basis.

## Abbreviations

GWA: Genome-wide association
IDEFICS: Identification and prevention of Dietary- and lifestyle-induced health EFfects In Children and infantS
