# Supplementary material for: Genetic associations vary across the spectrum of fasting serum insulin: results from the European IDEFICS/I.Family children’s cohort
Source: Diabetologia. 2023 Jul 7;66(10):1914–24. doi: 10.1007/s00125-023-05957-w (PMC10473990; doi:10.1007/s00125-023-05957-w)
Supplement: Supplementary file 1 — Supplementary file1 (PDF 1161 KB) [file 125_2023_5957_MOESM1_ESM.pdf]

Electronic Supplementary Material (ESM)

ESM Table 1: Coefficient of determination (adjusted R^2 in %) for quantile regression models for log insulin with and without further adjustment for genotypes identified in GWA analyses of specific percentile ranks, P15, P25, P50, P75, and P85 (Table 2) [a]

		Models with genotypes identified in GWA analyses of specific age- and sex-specific percentiles						
Quantile level	Model without genotypes	P15	P25	P50	P75	P85	Model with all genotypes	Δ R^2 (%) [b]
0.05	26.1	35.9	33.3	31.9	32.8	31.5	54.9	28.8
0.10	24.1	34.2	31.4	30.7	31.1	29.7	52.9	28.8
0.15	22.8	32.9	29.7	29.6	30.1	28.4	51.7	28.9
0.20	22.5	32.1	29.3	29.2	29.6	28.1	50.8	28.3
0.25	22.4	31.7	29.1	29.2	29.6	28.2	50.4	28.0
0.30	22.3	31.4	28.7	29.1	29.5	28.0	50.1	27.8
0.35	22.1	31.1	28.2	29.0	29.4	28.0	49.8	27.7
0.40	21.9	30.9	27.9	29.0	29.4	27.9	49.7	27.8
0.45	22.0	30.8	27.9	29.0	29.4	27.9	49.6	27.6
0.5	22.1	30.7	27.8	29.1	29.5	28.0	49.7	27.6
0.55	22.3	30.9	27.7	29.3	29.8	28.3	49.7	27.4
0.60	22.5	31.0	27.7	29.3	30.0	28.5	49.7	27.2
0.65	22.5	31.1	27.7	29.3	30.1	28.6	49.6	27.1
0.70	22.8	31.3	27.9	29.3	30.3	28.9	49.7	26.9
0.75	23.2	31.7	28.2	29.5	30.6	29.3	49.8	26.6
0.80	23.5	31.8	28.5	29.6	30.9	29.6	49.9	26.4
0.85	23.6	31.9	28.6	29.8	31.3	30.1	50.2	26.6
0.90	23.8	32.1	28.8	30.0	31.6	30.3	50.6	26.8
0.95	24.2	32.8	29.3	30.7	32.2	30.9	51.5	27.3

[a] all models adjusted for age, age^2, sex, BMI, country, PCs (background model); adjusted R^2 calculated following Koencker and Machado [1]

[b] difference in adjusted R^2 between a model including all genotypes (column 8) and the background model without genotypes (column 2)

ESM Table 2: Replication study of published associations in the IDEFICS/I.Family cohort (*n* = 2825)

			Published results for log-insulin				IDEFICS/I.Family results [a]			
									Effect size (SE) for log-insulin	
Chr	SNP	Nearest gene	Alleles (effect/other)	f	Effect size (SE)	Ref.	Effect allele	f	basic model	adjusted for BMI
1	rs2820436	*LYPLAL1*	C/A	0.67	0.02 (0.003)	[2]	A	0.30	-0.018 (0.019)	-0.013 (0.017)
1	rs2785980	*LYPLAL1*	T/C	0.67	0.016 (0.003) [b]	[3]	C	0.30	0.000 (0.019)	0.003 (0.017)
1	rs4846565	*LYPLAL1*	G/A	0.67	0.01 (0.002) [c]	[2]	A	0.30	-0.008 (0.019)	-0.003 (0.017)
1	rs6674544	*LYPLAL1*	A/G	0.57	0.018 (0.002)	[4]	G	0.36	-0.008 (0.018)	0.001 (0.017)
2	rs780094	*GCKR*	C/T	0.62	0.032 (0.004)	[5]	T	0.44	0.001 (0.017)	0.011 (0.016)
2				0.61	0.02 (0.003)	[3]				
2	rs1260326	*GCKR*	T/C	0.39	-0.023 (0.002)	[4]	T	0.44	0.004 (0.018)	0.010 (0.016)
2	rs1530559	*YSK4*	A/G	0.52	0.01 (0.003)	[2]	A	0.39	0.025 (0.021)	0.019 (0.019)
2	rs10195252	*GRB14*	T/C	0.59	0.02 (0.003)	[2]	C	0.41	-0.006 (0.017)	-0.017 (0.016)
2	rs7607980	*COBLL1, GRB14*	T/C	0.88	0.03 (0.003) [b]	[3]	C	0.13	0.007 (0.025)	-0.002 (0.023)
2	rs7565117	*COBLL1*	C/G	0.88	0.028 (0.003)	[4]	G	0.13	0.006 (0.026)	-0.004 (0.024)
2	rs13389219	*COBLL1*	T/C	0.39	-0.020 (0.002)	[4]	G	0.40	-0.004 (0.017)	-0.015 (0.016)
2	rs2943634	*IRS1*	C/A	0.66	0.02 (0.003) [b]	[3]	A	0.35	-0.017 (0.018)	-0.015 (0.017)
2	rs2943645	*IRS1*	T/C	0.63	0.02 (0.002)	[2]	C	0.37	-0.023 (0.018)	-0.021 (0.016)
2	rs2972143	*IRS1*	G/A	0.62	0.01 (0.003)	[2]	A	0.36	-0.025 (0.018)	-0.022 (0.016)
2	rs2943646	*LOC646736*	A/G	0.37	-0.025 (0.002)	[4]	A	0.37	-0.023 (0.018)	-0.021 (0.016)
3	rs17036328	*PPARG*	T/C	0.86	0.02 (0.003) [c]	[2]	C	0.13	-0.004 (0.026)	-0.011 (0.024)
3	rs1801282	*PPARG*	C/G	0.86	0.022 (0.004) [c]	[3]	G	0.13	-0.001 (0.026)	-0.008 (0.024)
3	rs35000407	*PPARG*	T/G	0.86	0.026 (0.003)	[4]	G	0.12	-0.004 (0.026)	-0.009 (0.024)
3	rs308971	*SYN2*	A/G	0.87	-0.022 (0.003)	[4]	G	0.11	0.017 (0.027)	0.026 (0.025)
3	rs17331151	*ITIH3*	T/C	0.11	-0.016 (0.003)	[4]	T	0.10	-0.011 (0.028)	-0.014 (0.026)
3	rs11708067	*ADCY5*	A/G	0.78	-0.014 (0.002)	[4]	G	0.20	-0.029 (0.021)	-0.030 (0.020)
3	rs62271373	*LINC01214*	A/T	0.06	0.026 (0.005)	[4]	A	0.04	-0.005 (0.043)	-0.003 (0.040)
4	rs11727676	*HHIP*	T/C	0.91	-0.020 (0.004)	[4]	C	0.10	-0.035 (0.028)	-0.026 (0.026)
4	rs3822072	*FAM13A*	A/G	0.48	0.01 (0.002)	[2, 6]	A	0.49	0.006 (0.017)	-0.001 (0.016)
4	rs3775380	*FAM13A*	A/G	0.50	-0.012 (0.002)	[4]	A	0.49	-0.014 (0.017)	-0.008 (0.016)
4	rs974801	*TET2*	G/A	0.38	0.01 (0.002) [c]	[2]	G	0.40	0.000 (0.017)	0.002 (0.016)

4	rs9884482	*TET2*	C/T	0.39	0.02 (0.002)	[2]	C	0.41	0.001 (0.017)	0.001 (0.016)
4			C/T	0.39	0.013 (0.002)	[4]				
4	rs4691380	*PDGFC*	C/T	0.67	0.02 (0.003) [b]	[3]	T	0.36	-0.006 (0.018)	0.001 (0.016)
4	rs6822892	*PDGFC*	A/G	0.68	0.01 (0.002) [c]	[2]	C	- [d]	-	-
4	rs6855363	*PDGFC*	T/C	0.68	0.013 (0.002)	[4]	C	0.36	-0.006 (0.018)	0.002 (0.016)
**5**	**rs4865796**	***ARL15***	**A/G**	**0.67**	**0.01 (0.003)**	**[2]**	**G**	**0.31**	**-0.035 (0.018)***	**-0.031 (0.017)**
				**0.68**	**0.017 (0.002)**	**[4]**				
5	rs459193	*ANKRD55, MAP3K1*	G/A	0.73	0.01 (0.002) [c]	[2]	A	0.28	0.003 (0.019)	-0.014 (0.017)
5	rs459193	*LOC101928448*	A/G	0.27	-0.018 (0.002)	[4]				
5	rs3936511	*LOC101928448*	A/G	0.82	-0.019 (0.003)	[4]	G	0.18	-0.015 (0.022)	-0.004 (0.020)
5	rs7708285	*ZBED3-AS1*	A/G	0.73	-0.013 (0.002)	[4]	G	0.28	-0.006 (0.019)	-0.013 (-0.017)
6	rs6912327	*UHRF1BP1*	T/C	0.80	0.02 (0.003) [c]	[2]	C	0.23	-0.007 (0.021)	-0.019 (0.019)
6	rs4646949	*UHRF1BP1*	T/G	0.75	0.017 (0.003) [b]	[3]	G	0.29	0.008 (0.019)	-0.012 (0.018)
6	rs2745353	*RSPO3*	T/C	0.51	0.01 (0.002)	[2]	T	0.49	0.009 (0.017)	0.008 (0.016)
6	rs116141873	*C6orf1*	T/G	0.04	0.043 (0.006)	[4]	T	0.03	-0.032 (0.051)	-0.028 (0.047)
6	rs2780215	*RPS10-NUDT3; NUDT3*	A/G	0.96	0.039 (0.006)	[4]	G	0.05	0.061 (0.040)	0.051 (0.037)
6	rs998584	*VEGFA*	A/C	0.49	0.012 (0.002)	[4]	A	0.48	-0.014 (0.017)	-0.016 (0.016)
6	rs9472135	*LINC01512*	T/C	0.70	0.011 (0.002)	[4]	C	0.31	0.014 (0.018)	0.006 (0.017)
6	rs1474696	*RSPO3*	A/G	0.49	-0.015 (0.002)	[4]	G	0.49	0.006 (0.017)	0.005 (0.016)
6	rs73013411	*QKI*	A/C	0.13	-0.018 (0.003)	[4]	A	0.13	-0.012 (0.026)	-0.010 (0.024)
7	rs1167800	*HIP1, POM121C*	A/G	0.54	0.02 (0.003)	[2, 6]	G	0.41	0.005 (0.017)	0.014 (0.016)
7	rs2108349	*GRB10*	A/G	0.66	-0.012 (0.002)	[4]	G	0.28	0.018 (0.019)	0.021 (0.018)
7	rs972283	*KLF14*	A/G	0.47	-0.011 (0.002)	[4]	A	0.47	-0.011 (0.017)	-0.008 (0.016)
7	rs7798471	*ZNF12*	C/T	0.27	Men: 0.007 (0.005)	[7]	C	0.27	0.008 (0.027)	0.005 (0.025)
7					Women: 0.026 (0.005)				-0.037 (0.027)	-0.015 (0.025)
8	rs983309	*PPP1R3B*	T/G	0.12	0.03 (0.004)	[2]	T	0.10	-0.001 (0.029)	0.014 (0.027)
8	rs4841132	*PPP1R3B*	A/G	0.10	0.025 (0.005) [b]	[3]	A	0.09	0.003 (0.032)	0.016 (0.029)
8	rs2126259	*PPP1R3B*	T/C	0.11	0.02 (0.003) [c]	[2]	T	0.09	0.007 (0.031)	0.025 (0.028)
8	rs330945	*PPP1R3B*	T/C	0.63	0.014 (0.002)	[4]	C	0.35	-0.021 (0.022)	-0.018 (0.021)
**8**	**rs7012814**	***LOC157273***	**A/G**	**0.48**	**-0.022 (0.002)**	**[4]**	**A**	**0.46**	**-0.048 (0.020)***	**-0.056 (0.019)****
8	rs4841132	*LOC157273*	A/G	0.11	0.026 (0.003)	[4]	A	0.08	0.003 (0.031)	0.016 (0.029)
8	rs13258890	*NKX2-6*	T/C	0.77	0.013 (0.003)	[4]	C	0.24	-0.028 (0.020)	-0.308 (0.019)
9	rs75179845	*ABO*	T/C	0.91	-0.022 (0.004)	[4]	C	0.09	0.034 (0.029)	0.036 (0.027)

10	rs7903146	*TCF7L2*	C/T	0.72<br>0.73<br>0.73	0.02 (0.003)<br>0.02 (0.004)<br>0.012 (0.002)	[2]<br>[3]<br>[4]	T	0.31	-0.011 (0.019)	-0.004 (0.017)
10	rs12243326	*TCF7L2*	T/C	0.75	0.02 (0.004)	[3]	C	0.29	-0.003 (0.019)	0.004 (0.018)
10	rs118164457	*PTEN*	T/C	0.96	-0.035 (0.006)	[4]	C	0.02	0.078 (0.065)	0.080 (0.060)
11	rs2845885	*MACROD1*	T/C	0.93	-0.020 (0.004)	[4]	C	0.05	-0.033 (0.039)	-0.017 (0.036)
12	rs35767	*IGF1*	A/G	0.82	0.02 (0.004)	[3]	A	0.17	-0.010 (0.023)	0.004 (0.021)
12	rs1402013	*IGF1*	A/G	0.35	-0.009 (0.002)	[4]	A	0.33	-0.001 (0.018)	-0.013 (0.017)
12	rs860598	*IGF1*	A/G	0.82	0.018 (0.003)	[4]	G	0.17	0.003 (0.022)	0.020 (0.021)
**12**	**rs6487237**	***GYS2***	**A/C**	**0.78**	**0.015 (0.003)**	**[4]**	**C**	**0.25**	**-0.047 (0.020)***	**-0.040 (0.018)***
12	rs111264094	*HDAC7*	C/G	0.97	0.057 (0.009)	[4]	G	0.01	0.057 (0.074)	0.033 (0.069)
12	rs1351394	*HMGA2*	T/C	0.49	-0.011 (0.002)	[4]	T	0.46	-0.013 (0.017)	-0.021 (0.016)
12	rs7133378	*DNAH10*	A/G	0.32	-0.013 (0.002)	[4]	A	0.32	-0.009 (0.018)	-0.014 (0.017)
16	rs1421085	*FTO*	C/T	0.42	0.02 (0.003)	[2]	C	0.43	0.028 (0.017)	-0.001 (0.016)
18	rs28671200	*C18orf25*	T/G	0.68	0.009 (0.002)	[4]	G	0.26	0.015 (0.020)	0.027 (0.019)
**18**	**rs12454712**	***BCL2***	**T/C**	**0.58**	**0.014 (0.003)**	**[4]**	**C**	**0.37**	**-0.019 (0.018)**	**-0.034 (0.016)***
**19**	**rs731839**	***PEPD***	**G/A**	**0.34**<br>**0.34**	**0.02 (0.003)**<br>**0.012 (0.002)**	**[2]**<br>**[4]**	**G**	**0.35**	**0.025 (0.018)**	**0.034 (0.017)***
20	rs1206760	*EYA2*	A/G	0.54	-0.011 (0.002)	[4]	G	0.43	-0.003 (0.018)	-0.002 (0.016)
22	rs39713	*MTMR3*	T/C	0.09	-0.017 (0.003)	[4]	T	0.06	-0.039 (0.036)	-0.016 (0.033)

[a] Linear regression of log insulin on genetic variants adjusted for age, sex, country, principal components, and survey

[b] GWAS included interactions with body mass index (BMI) (ns), mean beta coefficient at BMI = 25 and 30 units reported

[c] adjusted for BMI

[d] no variation for this variant detected in the IDEFICS/I.Family cohort

ESM Fig. 1: Methodological flow chart

① *Which genetic variants are associated with different regions of the age- and sex-specific insulin distribution?*

Genome-wide logistic regression analyses for five specific percentile ranks of *z*-insulin:
P15 (*n* = 402), P25 (*n* = 644), P50 (*n* = 1570), P75 (*n* = 877), P85 (*n* = 573)

↓

② *How do the associations with genetic variants identified above vary across the insulin spectrum?*

Quantile regression plots for log-insulin and individual genetic variants identified in ① for quantile levels ranging from 0.05 to 0.95 (binsize = 0.05)

↓

③ *How much of the total variation in insulin is explained by groups of variants associated with age- and sex-specific insulin percentiles?*

Quantile regression of log-insulin on groups of genetic variants associated with P15, P25, P50, P75, and P85, respectively, for quantile levels ranging from 0.05 to 0.95 (binsize = 0.05)

↓

④ *How do the associations with genetic variants identified above vary across the insulin spectrum?*

Linear regression of log-insulin on individual genetic variants identified in ①

ESM Fig. 2: Manhattan plot illustrating GWA results for the 15th, 25th, 50th, and 75th percentile ranks of the z-insulin distribution

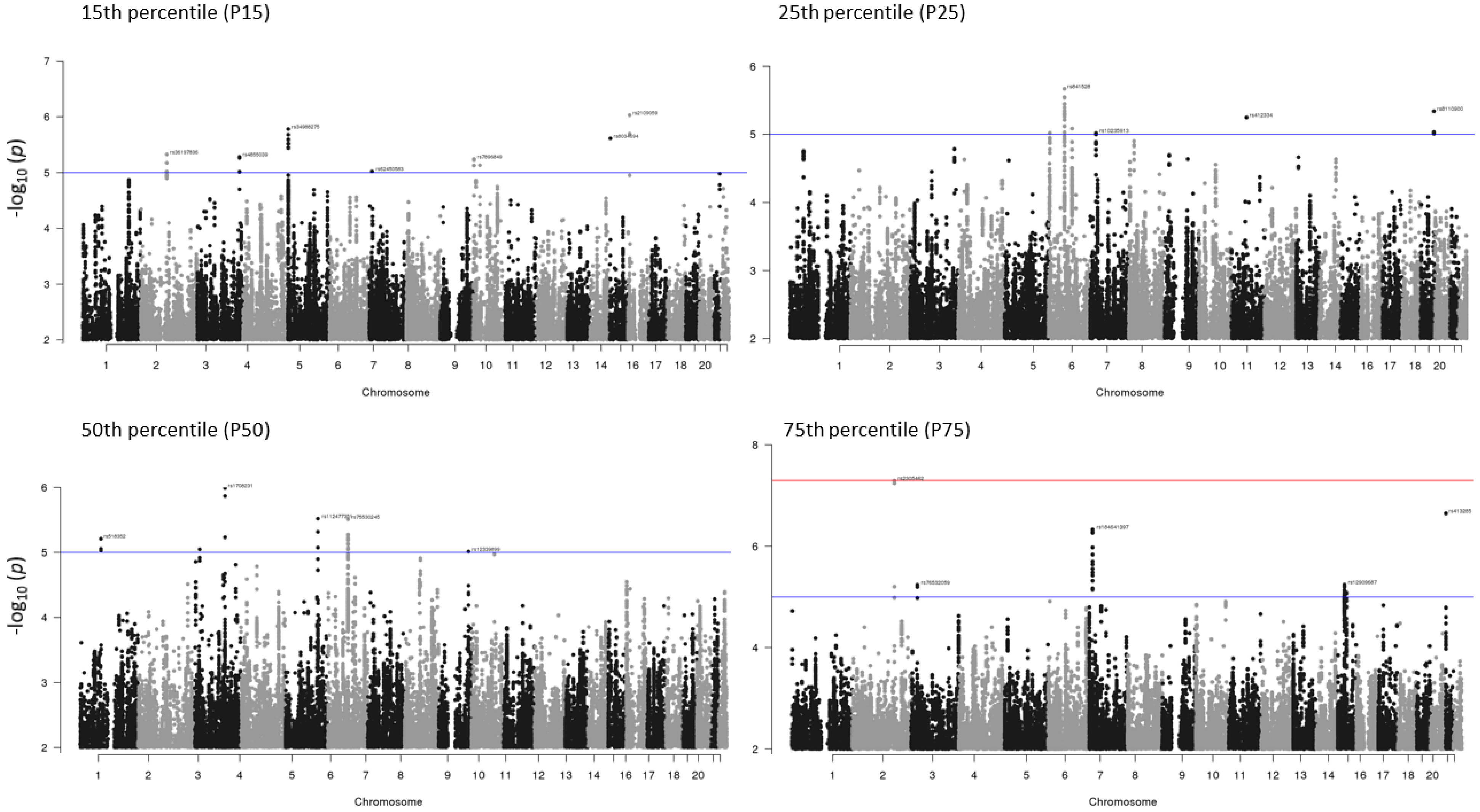

ESM Fig. 3: Quantile process plots for individual SNPs as listed in Table 2 including a test for heteroscedasticity across quantile levels (p_{hs} value). Quantile regression of log-insulin was performed for each SNP and adjusted for age, age^2, sex, BMI, country, principal components (regression parameters with 95% confidence bands).

Outcome for GWA analyses: 15th percentile rank (P15)

p_{hs} = 0.0002

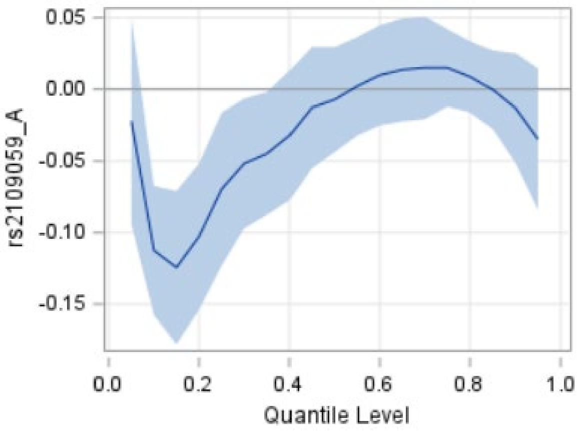

p_{hs} = 0.08

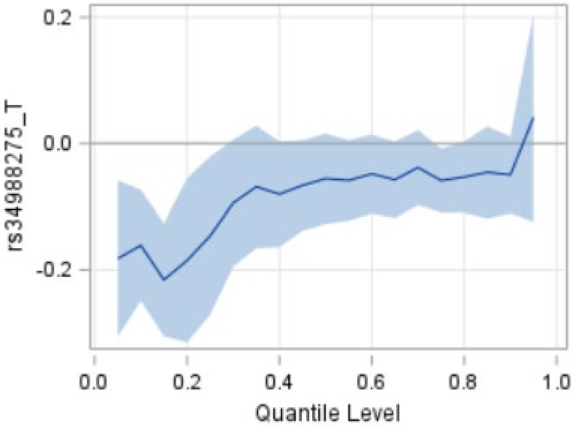

p_{hs} = 0.003

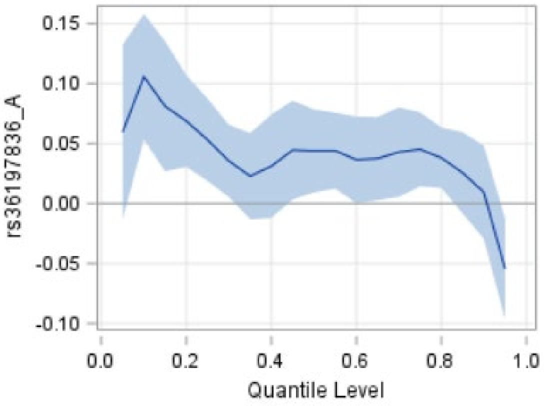

p_{hs} <.0001

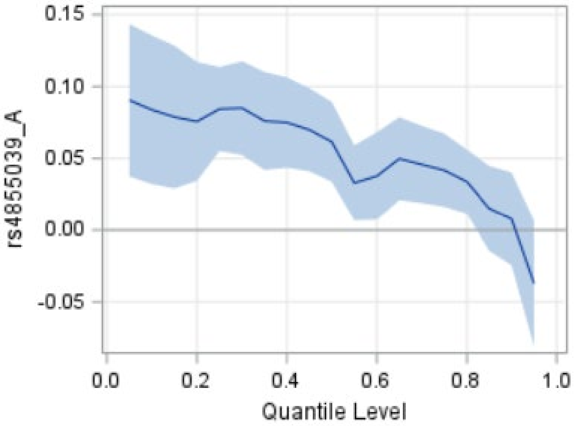

p_{hs} = 0.06

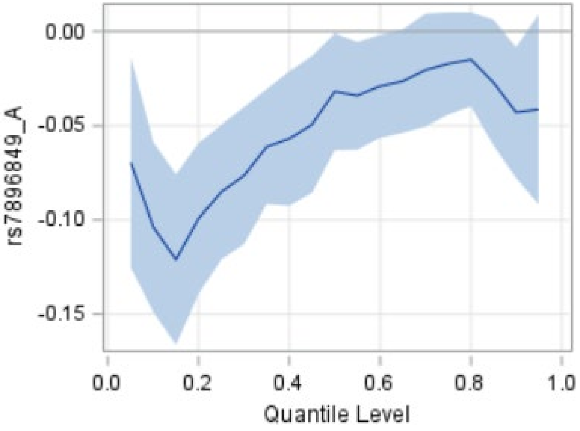

p_{hs} = 0.01

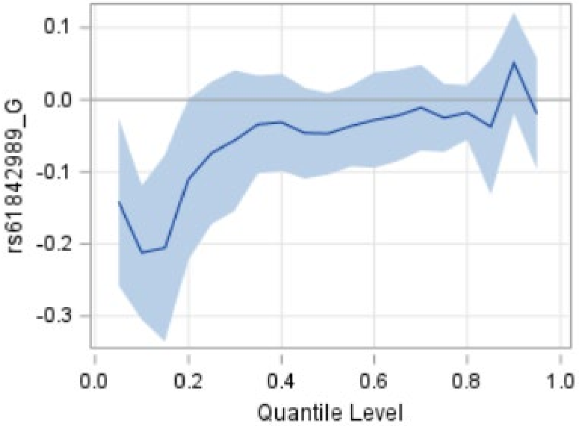

Outcome for GWA analyses: 25th percentile rank (P25)

p_{hs} = 0.0004

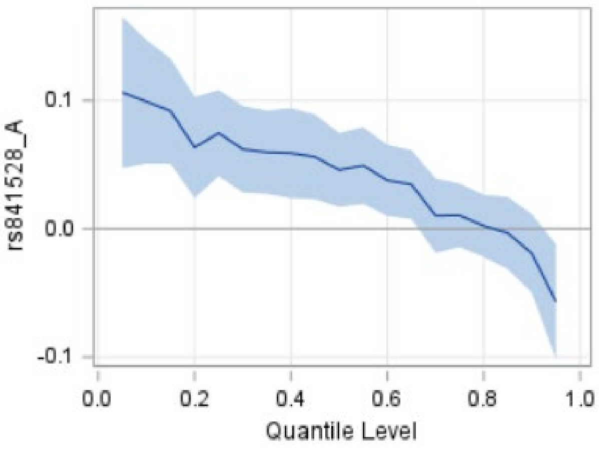

p_{hs} = 0.009

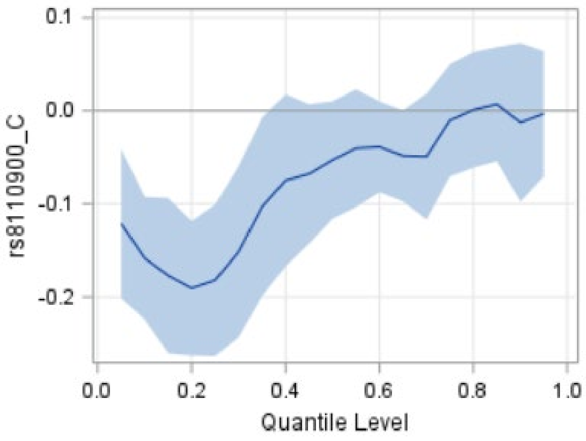

p_{hs} = 0.004

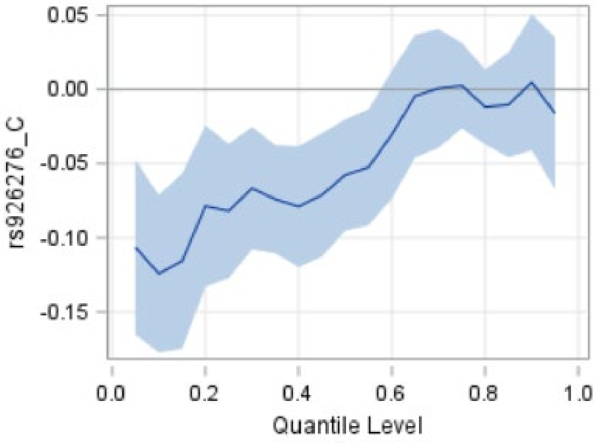

p_{hs} = 0.06

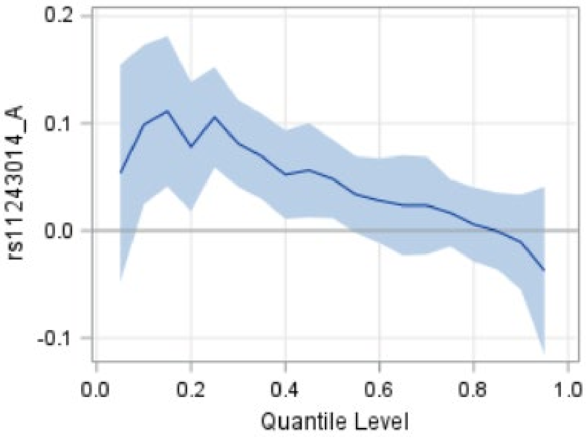

Outcome for GWA analyses: 50th percentile rank (P50, median rank)

p_{hs} = 0.2

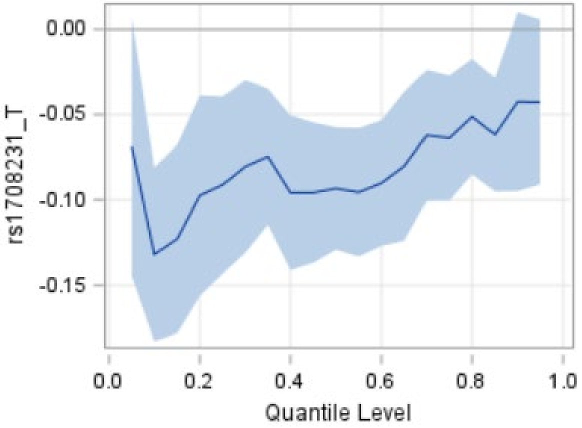

p_{hs} = 0.01

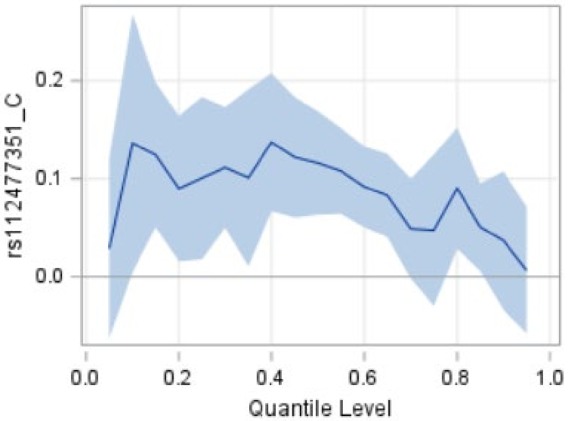

p_{hs} = 0.01

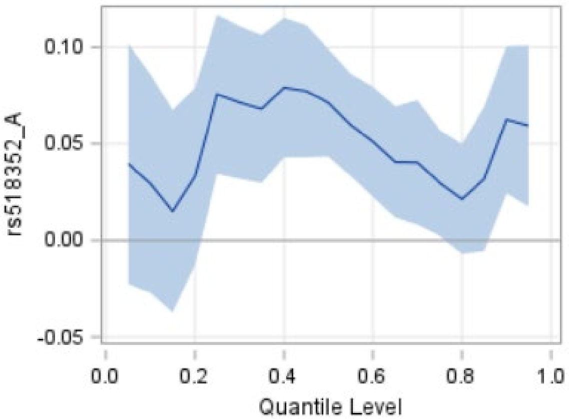

p_{hs} = 0.03

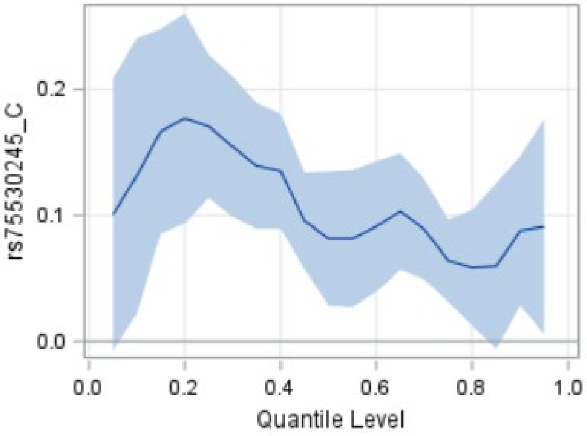

p_{hs} = 0.07

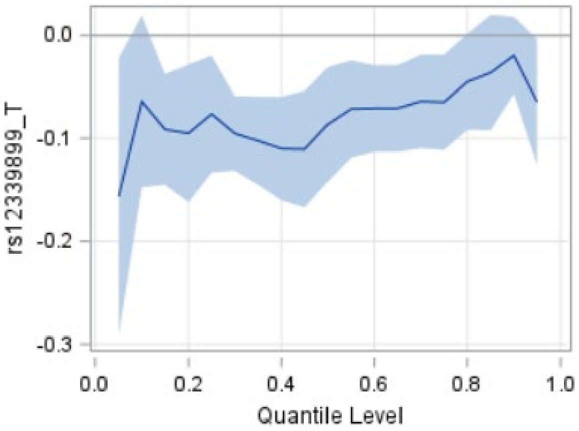

Outcome for GWA analyses: 75th percentile rank (P75)

p_{hs} = 0.5

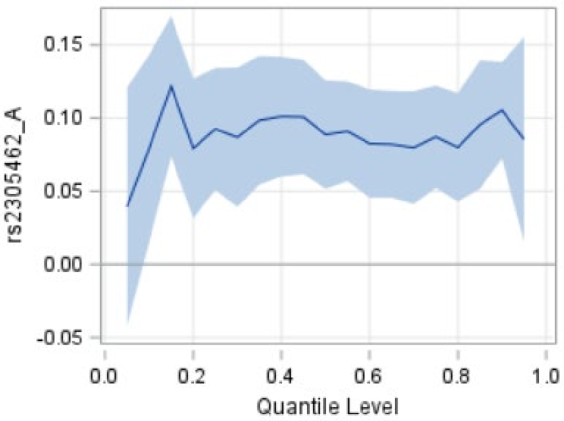

p_{hs} = 0.3

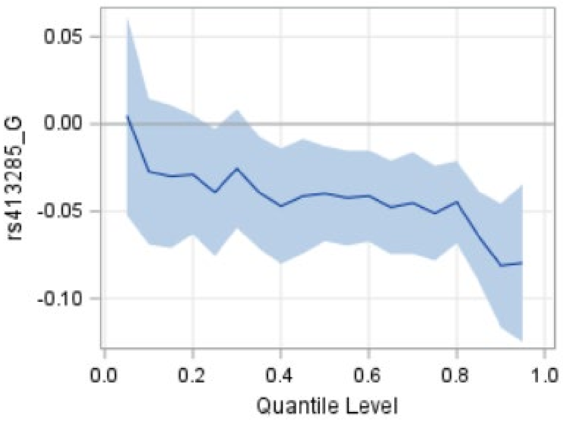

p_{hs} = 0.15

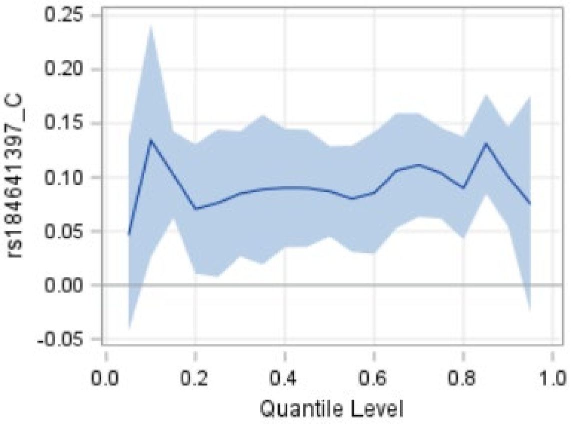

p_{hs} = 0.15

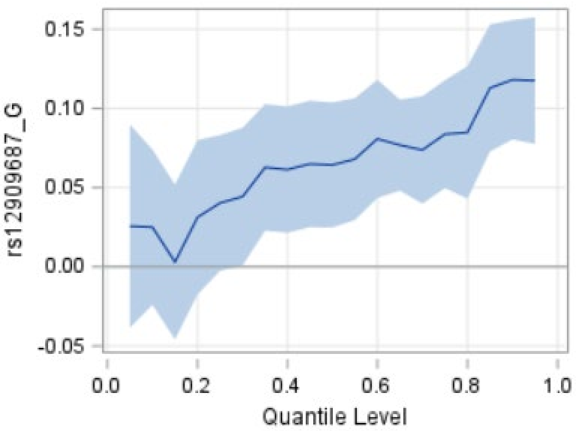

p_{hs} = 0.2

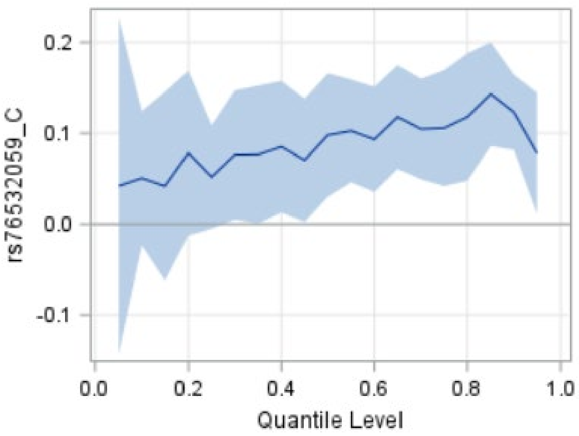

Outcome for GWA analyses: 85th percentile rank (P85)

p_{hs} = 0.01

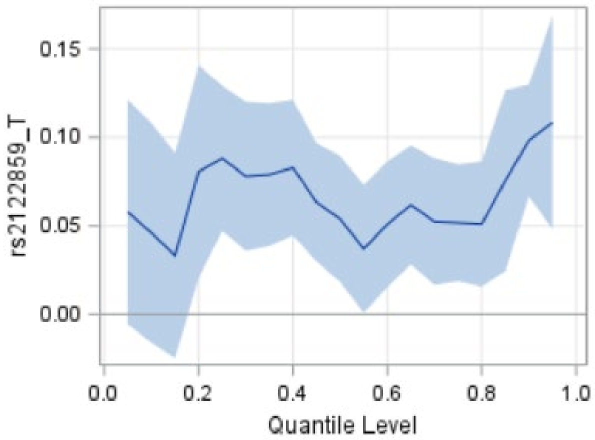

p_{hs} = 0.003

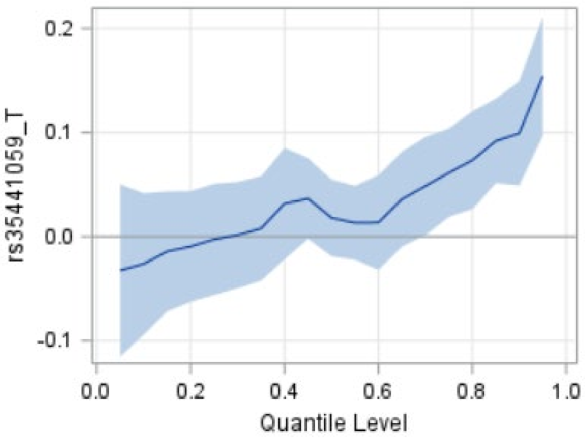

p_{hs} = 0.15

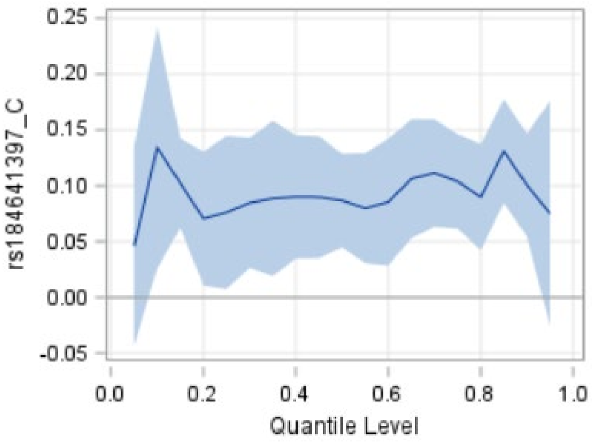

p_{hs} = 0.05

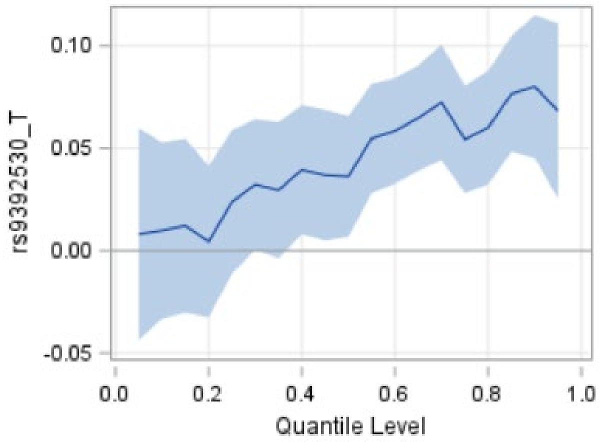

p_{hs} = 0.02

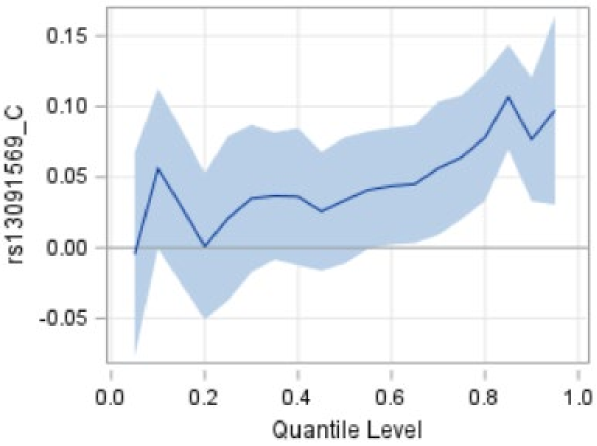

APOE-4 alleles

p_{hs} = 0.17 (not adjusted for BMI)

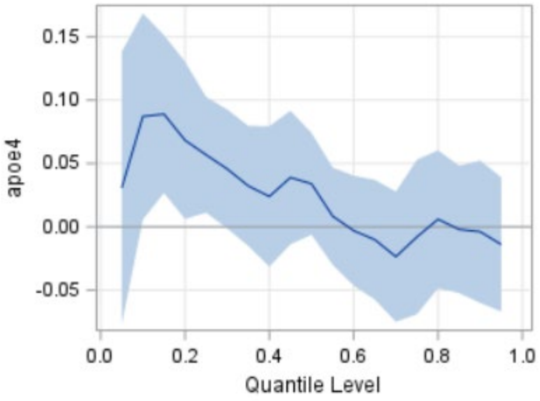

p_{hs} = 0.4 (adjusted for BMI)

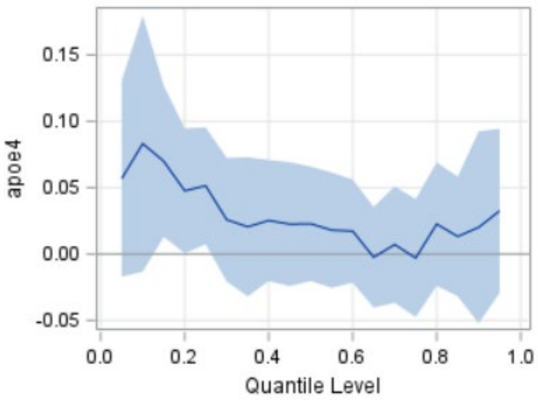

ESM Fig. 4: Quantile plots for selected SNPs from ESM Tab. 2

rs4865796, *ARL15*

without adjustment for BMI, p_{hs} = 0.004

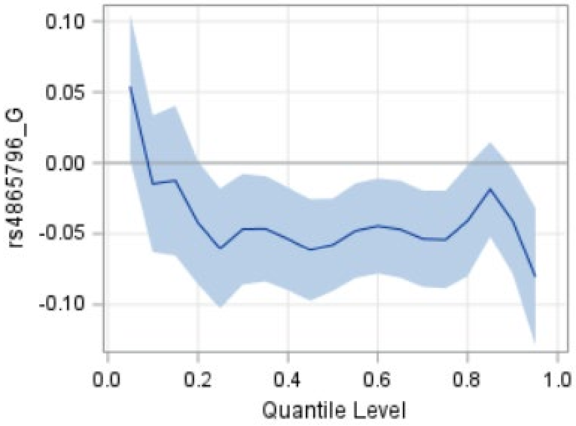

further adjusted for BMI, p_{hs} = 0.2

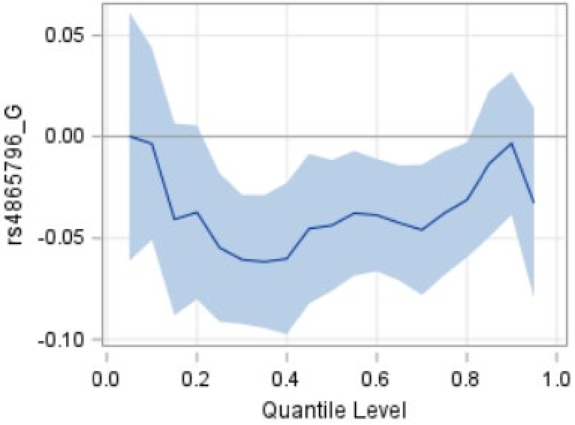

rs7012814, *LOC157273*

without adjustment for BMI, p_{hs} = 0.3

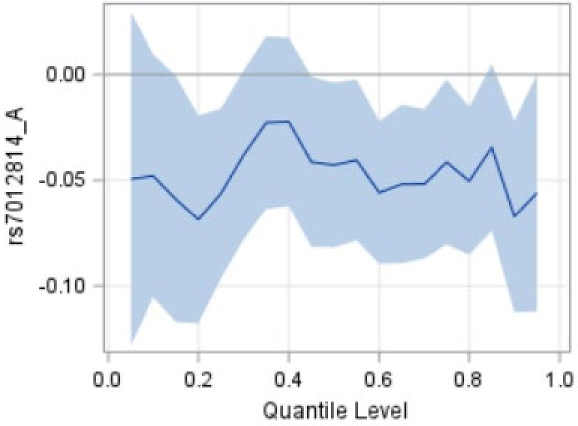

further adjusted for BMI, p_{hs} = 0.3

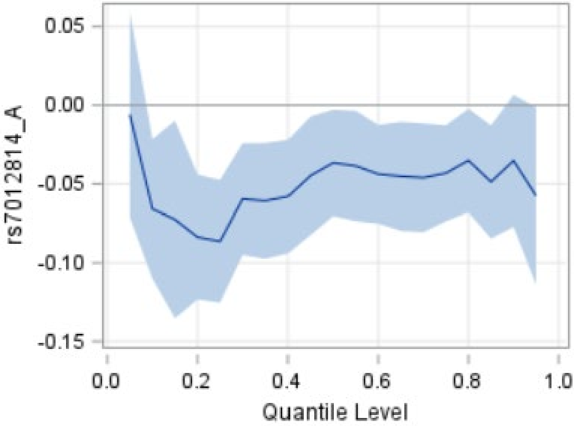

rs6487237, *GYS2*

without adjustment for BMI, p_{hs} = 0.9

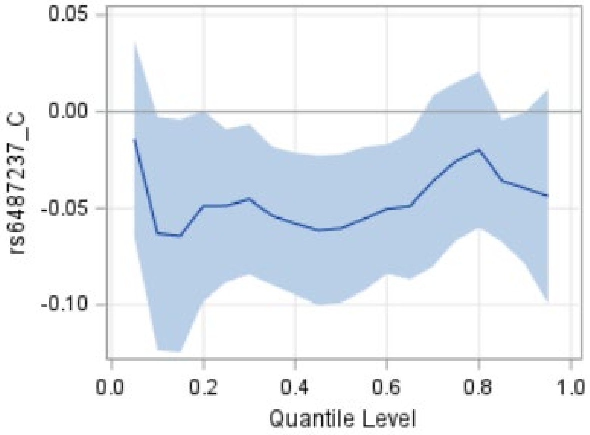

further adjusted for BMI, p_{hs} = 0.6

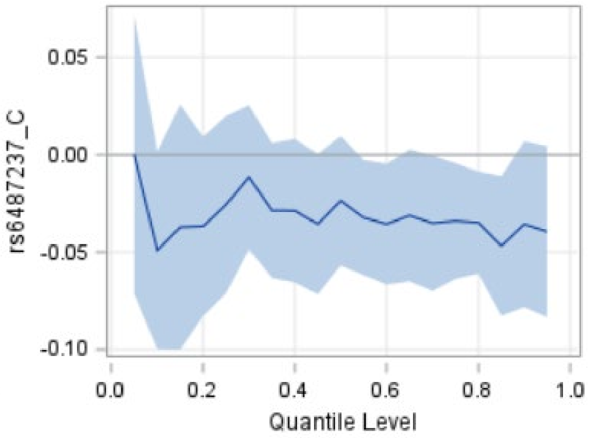

rs12454712, *BCL2*

without adjustment for BMI, p_{hs} = 0.9

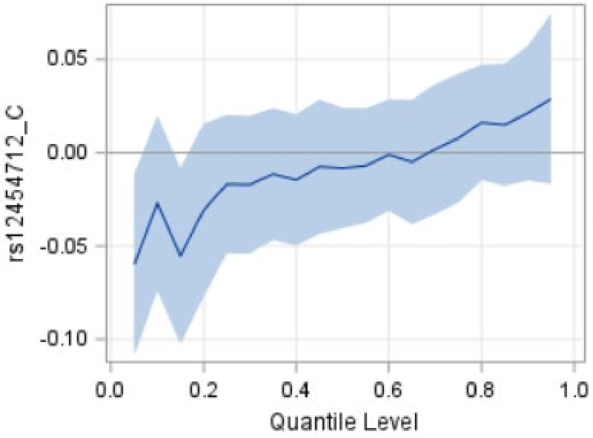

further adjusted for BMI, p_{hs} = 0.3

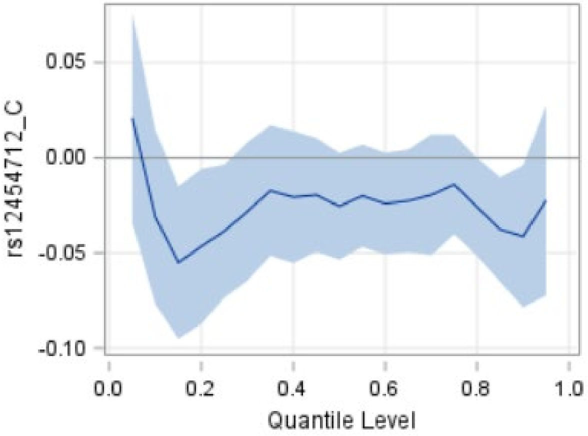

rs731839, *PEPD*

without adjustment for BMI, p_{hs} = 0.8 further adjusted for BMI, p_{hs} = 0.3

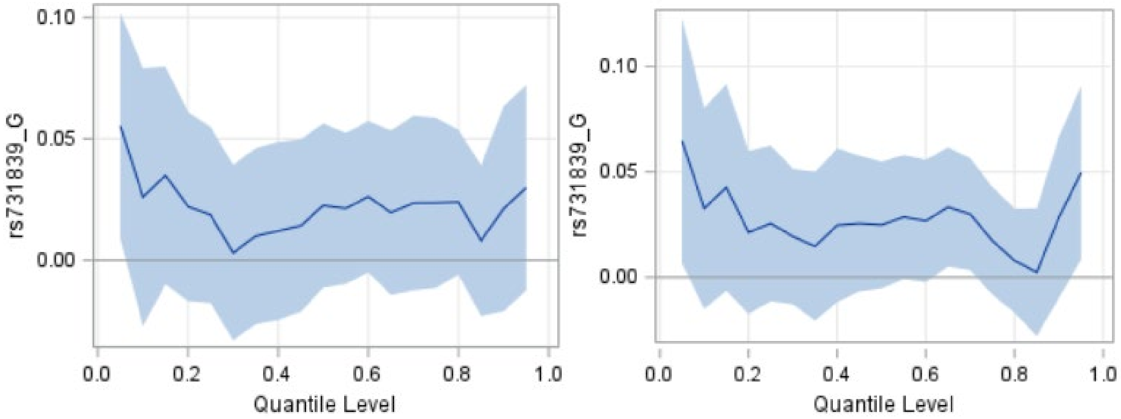

References

[1] Koenker R, Machado JAF (1999) Goodness of fit and related inference processes for quantile regression. Journal of the American Statistical Association 94(448): 1296-1310. Doi 10.2307/2669943

[2] Scott RA, Lagou V, Welch RP, et al. (2012) Large-scale association analyses identify new loci influencing glycemic traits and provide insight into the underlying biological pathways. Nat Genet 44(9): 991-1005. 10.1038/ng.2385

[3] Manning AK, Hivert MF, Scott RA, et al. (2012) A genome-wide approach accounting for body mass index identifies genetic variants influencing fasting glycemic traits and insulin resistance. Nat Genet 44(6): 659-669. 10.1038/ng.2274

[4] Chen J, Spracklen CN, Marenne G, et al. (2021) The trans-ancestral genomic architecture of glycemic traits. Nat Genet 53(6): 840-860. 10.1038/s41588-021-00852-9

[5] Dupuis J, Langenberg C, Prokopenko I, et al. (2010) New genetic loci implicated in fasting glucose homeostasis and their impact on type 2 diabetes risk. Nat Genet 42(2): 105-116. 10.1038/ng.520

[6] Lundback V, Kulyte A, Strawbridge RJ, et al. (2018) FAM13A and POM121C are candidate genes for fasting insulin: functional follow-up analysis of a genome-wide association study. Diabetologia 61(5): 1112-1123. 10.1007/s00125-018-4572-8

[7] Lagou V, Magi R, Hottenga JJ, et al. (2021) Sex-dimorphic genetic effects and novel loci for fasting glucose and insulin variability. Nat Commun 12(1): 24. 10.1038/s41467-020-19366-9
